# Expected Health Benefits as the Ultimate Outcome of Information Available on Stroke Engine, a Knowledge Translation Stroke Rehabilitation Website: Web-Based Survey

**DOI:** 10.2196/44715

**Published:** 2023-05-08

**Authors:** Annie Rochette, Aliki Thomas, Nancy M Salbach, Brigitte Vachon, Anita Menon, Lise Poissant, Maurane Boutin, Roland Grad, Pierre Pluye

**Affiliations:** 1 Centre for Interdisciplinary Research in Rehabilitation of Greater Montreal Montreal, QC Canada; 2 School of Rehabilitation Université de Montréal Montreal, QC Canada; 3 School of Physical and Occupational Therapy McGill University Montreal, QC Canada; 4 Institute for Health Sciences Education McGill University Montreal, QC Canada; 5 Department of Physical Therapy Rehabilitation Sciences Institute University of Toronto Toronto, ON Canada; 6 The KITE Research Institute University Health Network Toronto, ON Canada; 7 Centre de recherche Fernand Séguin Montreal, QC Canada; 8 Department of Family Medicine McGill University Montreal, QC Canada

**Keywords:** crowdsourcing, health-related information, internet, knowledge translation, rehabilitation, stroke

## Abstract

**Background:**

Electronic knowledge resources are readily available and typically target different audiences, including health professionals and the public, that is, those with lived experience and their relatives. The knowledge-to-action framework, in combination with the information assessment method (IAM), considering both the *value-of-information* construct and the conceptual model of *acquisition-cognition-application*, can be used to support the evaluation process of such resources. As an example, Stroke Engine is an evidence-based knowledge translation resource in stroke rehabilitation (assessments and interventions) for health professionals and students as well as individuals who have sustained a stroke and their relatives. According to Google Analytics, the website is perused >10,000 times per week.

**Objective:**

With the overall aim to improve the content available on Stroke Engine, we documented Stroke Engine users’ perceptions of situational relevance, cognitive impact, intention to use, and expected patient and health benefits regarding the information consulted.

**Methods:**

A web-based survey anchored in the IAM was made available via an invitation tab. The IAM is a validated questionnaire that is designed to assess the value of information. Sociodemographic characteristics were also collected, and a space for free-text comments was provided. Descriptive statistics were used, and thematic analysis was used for the free-text comments.

**Results:**

The sample consisted of 6634 respondents. Health professionals (3663/6634, 55.22%) and students (2784/6634, 41.97%) represented 97.18% (6447/6634) of the total responses. The remaining 2.82% (187/6634) of the responses were from individuals who had sustained a stroke (87/6634, 1.31%) and their relatives (100/6634, 1.51%). Regarding situational relevance, *assessments* (including selecting, obtaining, and interpreting results from a test) was the main topic searched by health professionals (1838/3364, 54.64%) and students (1228/2437, 50.39%), whereas general information on stroke rehabilitation was the top-ranked topic for nearly two-thirds of the individuals with stroke (45/76, 59%) and their relatives (57/91, 63%). Cognitive impact was characterized by *learning something new*. Intention to use was high (4572/6379, 71.67%) among the respondents and varied in context (eg, refine a topic, research, class assignments, teaching, and education). Respondents commented on ways to improve content. Expected patient and health benefits such as improvement in health and well-being was the top-ranked category for all 4 subgroups, followed by the avoidance of unnecessary or inappropriate treatment for health professionals (183/623, 29.4%) and a feeling of being reassured for individuals with stroke (26/75, 35%) and their relatives (28/97, 29%).

**Conclusions:**

Valuable feedback on Stroke Engine was obtained in terms of its accessibility, relevance for informational needs and retrieval, accuracy, and applicability; however, of utmost importance is the potential implementation of its evidence-based content in clinical practice and the perceived expected impact on patients, their relatives, and their health professionals. The feedback received allowed for corrections and the identification of key topics for further development.

## Introduction

### Background

In 2020, 92.3% of Canadians aged ≥15 years were internet users, including 77.6% of seniors (aged ≥65 years) [1]. Health information on the internet is readily available and typically targets different audiences such as health professionals and the public, that is, those with lived experience and their relatives. Indeed, electronic knowledge resources are now readily available and provide information about various health conditions [2,3]. Electronic knowledge resources can facilitate clinical decision-making, increase the understanding of disease of individuals with lived experience and their relatives, or override human memory [4]. In the form of texts, images, sounds, or videos, these resources can originate from different databases [5]; for example, *Google Scholar* is a Google service that can be used by the public to search for scientific articles, identifying those that are approved or not by a peer-review committee [6]. Similarly, *PubMed* is a searchable database that contains >34 million medical citations from scientific journals, web-based books, and biomedical literature. Considering the impact that this information can have on the users of these resources, the knowledge-to-action (KTA) model [7] argues that for this information to be considered a third generation of knowledge, it is essential that the information transmitted is not only reliable and valid but also synthesized in a way that is relevant, understandable, and readily applicable by end users (eg, health professionals and people with lived experience).

The Stroke Engine website [8] was built with the goal of contributing to bridging the gap between available research findings and their application in current clinical practice in stroke rehabilitation [9-13]. Indeed, with >1700 motor- and cognitive-based stroke rehabilitation randomized controlled trials published between 1972 and 2018 [14], stroke rehabilitation is an area where there is an abundance of available scientific evidence. This comprehensive site, available in English and French, includes the most current information about the effectiveness of various interventions in both scientific and lay-language format as well as the psychometric and pragmatic properties [15] of >100 stroke-related assessment tools used in stroke rehabilitation. Stroke Engine’s content is derived from multiple sources, including the Evidence-Based Review of Stroke Rehabilitation [16], and extensive reviews of databases such as MEDLINE, CINAHL, the Cochrane Library, HealthSTAR, Health and Psychosocial Instruments, CANCERLIT, and PsycINFO. The goal is to provide health professionals (physicians and clinicians working with individuals with stroke in any setting), students (in any discipline), and individuals with stroke and their relatives with evidence-based information on stroke rehabilitation. The website is led by the first author (AR), and its content relies on the expertise of a research team (including 5 coauthors [AT, NMS, BV, AM, and LP]). Indeed, a dedicated team of senior researchers, graduate students, and research assistants with expertise in specific areas also contribute to creating reviews for each topic and evaluating their quality. Contributing authors are listed on each page and topic along with the date of the last update.

The website is perused by >10,000 visitors per week. According to Google Analytics, the most popular pages are related to assessment, although the page *Find an intervention* is ranked in the top 10. The visitors can be health professionals, students, or individuals with stroke and their relatives. We wondered about what information they are searching for and what they think about what they find. Obtaining answers to these questions [17] is essential to present *better-than-best* evidence [18]. Indeed, 2-way knowledge translation assumes that information users have the expertise [19] to provide feedback on the relevance, accuracy, and applicability of the available information. Therefore, we created a knowledge translation resource that synthesizes information in a way that is relevant to end users as well as understandable and readily applicable by them. However, unless we apply a rigorous evaluation process, we do not know how this information is applied and whether it has the intended ultimate targeted benefits for health.

Despite the purpose and many benefits of internet resources, including Stroke Engine, it is unclear how the impact of its use by end users should be documented. We argue that outcomes such as internet access as well as information needs and retrieval, as documented in most studies [20,21], are insufficient, whereas the actual implementation in practice and health benefits are most relevant. The information assessment method (IAM) [22] can help to overcome these limitations because it is based on both the *value-of-information* construct and the conceptual model of *acquisition-cognition-application* [23], which was extended to 4 levels of outcomes: situational relevance, cognitive impact, intention to use, and expected patient and health benefits [24]. Thus, this brief, systematic web-based questionnaire can evaluate and document reflection on health information because it fosters reflective learning, evaluation, and 2-way knowledge translation [25].

### Research Questions and Objectives

Our research questions were as follows:

- Who are the visitors?
- Are they mostly health care professionals, students, and individuals with stroke and their relatives?
- What information are they searching for?
- What do they think about what they find?

The objective of this study was to document Stroke Engine users’ perceptions of (1) situational relevance, (2) cognitive impact, (3) intention to use, and (4) expected patient and health benefits regarding the information they consulted on the Stroke Engine website.

## Methods

### Study Design

As the Stroke Engine website is visited by approximately 500,000 individuals yearly, we relied on a crowdsourcing developmental evaluation [26,27], using a web-based survey to obtain feedback on its content. Crowdsourcing is defined as “the practice of obtaining needed services, ideas, or content by soliciting contributions from a large group of people and especially from the online community rather than from traditional employees or suppliers” [28]. Crowdsourcing has been used by search engines such as Google to identify the most useful and most visited internet pages [29], and it has also been used to develop innovative learning networks such as Wikipedia [30,31].

### The KTA Cycle

We used the process depicted in the KTA cycle [7] to guide our plan to evaluate Stroke Engine, whereas crowdsourcing was used as a method of data collection for evaluating the website. As the Stroke Engine website disseminates the best available scientific evidence, we needed the survey information to understand and improve how it supports implementation of this evidence in practice and ultimately benefits individuals with stroke and their relatives. The Stroke Engine team members *synthesize the information* about stroke rehabilitation assessment and treatment interventions (corresponding to the knowledge creation funnel at the center of the KTA) and then post it on the website, which enables *diffusion* of information to a large international audience (corresponding to the action and application cycle of the KTA). Visitors or users of this knowledge tool (website) are invited to *assess the information* through a web-based survey built into the website (corresponding to the *evaluate outcomes* step of the KTA); data and feedback are analyzed, and the results are used to *improve content* on the website and help prioritize future content developments (corresponding to the *sustain knowledge use* step of the KTA).

### IAM Questionnaire

The web-based survey uses the IAM questionnaire developed by members of our research team (RG and PP) and follows the reasoned action approach [32]. The IAM is a validated method to assess the value of information in terms of its (1) situational relevance, (2) cognitive impact, (3) intention to use, and (4) expected patient health benefits [18,24]. Two versions of the IAM questionnaire were used: the IAM for clinicians and the IAM for patients and consumers. Both contain 5 questions that can be answered in <2 minutes. A space was provided for optional free-text comments. The items under each question were adapted to the context of stroke rehabilitation using a 2-phase process: consultation of stroke experts (n=5) using the nominal group method, followed by a consultation of users through 2 focus groups (1 with 6 clinicians and 1 with 3 individuals with stroke). Minor modifications were made to both versions of the questionnaire. For the IAM for clinicians, 2 questions were slightly modified, 15 items were modified, 4 were removed, and 3 were added in comparison with the initial version to clarify the statements. In the IAM for patients and consumers, 2 questions were modified to contextualize to stroke rehabilitation, 7 items were modified, and 4 items were added.

### Data Collection Procedures

An invitation tab was added to the right side of the website to invite users to a web-based survey using IAM. This method has been successfully used for >15 years in >25 projects, 4 countries, and with various health conditions [22]. Invitation tabs in English or French appeared on the respective language pages of the website. We added an invitation pop-up window that appeared when a user had been on the same page for >30 seconds because health professionals told us that they could not easily find the invitation tab on the right side of the website.

The survey was completed on an anonymous, voluntary basis. It was thus possible for respondents to complete the survey questionnaire more than once. Data collected between October 7, 2020, and May 25, 2021, were used for analysis. According to Google Analytics, for the period during which the survey data were collected, the majority of the visitors (206,017/243,628, 84.56%) came from organic search, 11.75% (28,632/243,628) landed directly, 4.99% (12,165/243,628) were referred, whereas others represented 1.15% (2795/243,628). Following the 5 questions of the IAM, we collected minimal sociodemographic data (eg, age, gender, education, and location) for descriptive purposes. As the survey questionnaire was built into the website, the system also allowed us to collect data regarding the specific page visited when the survey was completed.

### Ethics Approval, Informed Consent, and Participation

Respondents provided consent by agreeing to fill in the web-based questionnaire through the following text: “Thank you for your feedback which will be used to improve the website and prioritize future developments. All data are analyzed anonymously. By completing the survey and clicking on the submit button below, you are providing consent. Ethics approval was obtained from the health ethics board of the University of Montreal (Projet 17-157-CERES-D). For any questions, please contact the principal investigator [name and contact information].” There was no compensation for filling in the web-based questionnaire.

### Data Analysis

We used descriptive statistics (frequency and percentage) to describe feedback on the information consulted. Optional free-text comments were coded using thematic analysis [33]. We deliberatively chose to not perform a content analysis, which typically includes frequency of categories and themes [34], because comments were optional, and these were used in an exploratory manner to deepen our understanding of the answers to the IAM questionnaire. All free-text comments were uploaded into NVivo 10 (QSR International) with ID numbers and coded inductively by the first author with a tag relating to the meaning of the content. It was not possible to split the comments according to the type of respondent, but whenever the content of the comments related to an individual with stroke or their relative, these were tagged as such. In addition, we could retrieve respondent characteristics for a specific quote using the ID number. As such, comments were analyzed for the whole sample. Codes were then grouped according to major themes and are presented following the study objectives. Themes and related associated comments were reviewed by the research team.

## Results

### Sample Description

A total of 6634 completed questionnaires were available at the time of analysis (refer to Table 1 for sample description). Health professionals (3663/6634, 55.22%) and students (2784/6634, 41.97%) represented 97.18% (6447/6634) of the total responses. The remaining 2.82% (187/6634) of the responses were from individuals who had sustained a stroke (87/6634, 1.31%) and their relatives (100/6634, 1.51%). Nearly half of the respondents (3182/6518, 48.82%) were aged between 19 and 29 years. Among the health professionals and students, 77.15% (4822/6250) of the respondents were female. Almost all survey respondents (6027/6397, 94.22%) had completed a college degree or higher. The most common geographical locations of respondents were Western Europe (2406/6634, 37.45%) and North America (2203/6634, 34.29%), followed by Eastern Asia (422/6634, 6.57%) and Australia or New Zealand (353/6634, 5.49%). Tables 2 and 3 provide an overview of the descriptive results of the IAM for health professionals and students (Table 2) and for individuals with stroke and their relatives (Table 3). The main themes emerging from the free-text comments (n=950) for all respondents are presented in the subsections that are presented after the tables according to the study objectives. Regarding situational relevance, the main themes are summarized as 8 subthemes: assessment approach, how to obtain a test, interpretation of the test results, clinical decision-making, empowerment and coping, research purposes, resource for teaching, and educate and inform clients. The main cognitive impact was characterized by the following subtheme: learning something new. Intention to use was composed of 5 subthemes: refine knowledge on a topic with prior knowledge, intention to use a test and looking as to where to obtain it, use information in the context of a class assignment, visual or format and ease of finding needs improvements, and information insufficient and perceived as incomplete. Respondents also left comments identifying important topics to add. The main subtheme under the objective of expected patient and health benefits related to using an assessment to provide feedback on improvements. Respondents also used free-text comments to leave general comments that were overall positive.

**Table 1:** Respondents’ characteristics.

			Health professionals (n=3663)		Students (n=2784)		Individuals with stroke (n=87)		Relatives (n=100)		Total (N=6634)
**Age group (years)**											
	≤18, n (%)	21 (0.59)		73 (2.65)		0 (0)		1 (1.1)		95 (1.46)	
	19-29, n (%)	957 (26.72)		2221 (80.53)		1 (1)		3 (3.2)		3182 (48.82)	
	30-39, n (%)	1010 (28.2)		282 (10.22)		9 (11)		8 (8.5)		1309 (20.08)	
	40-49, n (%)	771 (21.52)		120 (4.35)		14 (17)		12 (12.8)		917 (14.07)	
	50-59, n (%)	560 (15.63)		42 (1.52)		21 (25)		27 (28.7)		650 (9.97)	
	60-69, n (%)	214 (5.97)		8 (0.29)		21 (25)		23 (24.5)		266 (4.08)	
	70-79, n (%)	31 (0.86)		5 (0.18)		13 (15)		16 (17)		65 (1)	
	≥80, n (%)	18 (0.5)		7 (0.25)		5 (6)		4 (4.3)		34 (0.52)	
	Missing, n	81		26		3		6		116	
**Sex**											
	Female, n (%)	2719 (77.09)		2103 (77.23)		41 (51)		58 (63)		4921 (76.63)	
	Male, n (%)	711 (20.16)		501 (18.4)		37 (46)		32 (34.8)		1281 (19.95)	
	Prefer not to answer, n (%)	97 (2.75)		119 (4.37)		2 (3)		2 (2.2)		220 (3.43)	
	Missing, n	136		61		7		8		212	
**Language of survey completion**											
	English, n (%)	2689 (73.41)		1823 (65.48)		71 (82)		78 (78)		4661 (70.26)	
	French, n (%)	974 (26.59)		961 (34.52)		16 (18)		22 (22)		1973 (29.74)	
**Level of education completed**											
	None, n (%)	5 (0.14)		5 (0.18)		0 (0)		0 (0)		10 (0.15)	
	Primary, n (%)	7 (0.2)		12 (0.44)		1 (1)		2 (2.2)		22 (0.34)	
	Secondary or high school, n (%)	17 (0.48)		287 (10.5)		23 (28)		11 (11.8)		338 (5.22)	
	College, n (%)	152 (4.26)		277 (10.13)		22 (27)		16 (17.2)		467 (7.21)	
	University undergraduate, n (%)	882 (24.69)		1559 (57.02)		16 (20)		21 (22.6)		2478 (38.23)	
	University postgraduate, n (%)	2461 (68.9)		561 (20.52)		18 (22)		42 (45.2)		3082 (47.55)	
	I do not know, n (%)	48 (1.34)		33 (1.21)		2 (2)		1 (1.1)		84 (1.3)	
	Missing, n	91		50		5		7		153	
**Location**											
	Western Europe, n (%)	1210 (34.04)		1145 (42.36)		25 (32)		26 (29.2)		2406 (37.45)	
	North America, n (%)	1365 (38.4)		743 (27.49)		47 (60)		48 (53.9)		2203 (34.29)	
	Eastern Asia, n (%)	197 (5.54)		220 (8.14)		3 (4)		2 (2.2)		422 (6.57)	
	Australia or New Zealand, n (%)	233 (6.55)		116 (4.29)		1 (1)		3 (3.4)		353 (5.49)	
	Central Asia, n (%)	118 (3.32)		101 (3.74)		0 (0)		2 (2.2)		221 (3.44)	
	Central or South America, n (%)	111 (3.12)		78 (2.89)		0 (0)		0 (0)		189 (2.94)	
	Eastern Europe, n (%)	75 (2.11)		57 (2.11)		0 (0)		1 (1.1)		133 (2.07)	
	Western Asia, n (%)	64 (1.8)		67 (2.48)		0 (0)		0 (0)		131 (2.04)	
	South Africa, n (%)	63 (1.77)		57 (2.11)		1 (1)		3 (3.4)		124 (1.93)	
	North Africa, n (%)	52 (1.46)		41 (1.52)		1 (1)		2 (2.2)		96 (1.49)	
	Pacific Ocean, n (%)	32 (0.9)		47 (1.73)		0 (0)		2 (2.2)		81 (1.26)	
	Indian Ocean, n (%)	16 (0.45)		24 (0.88)		0 (0)		0 (0)		40 (0.62)	
	Caribbean, n (%)	13 (0.36)		7 (0.26)		0 (0)		0 (0)		20 (0.31)	
	Central Africa, n (%)	6 (0.17)		0 (0)		0 (0)		0 (0)		6 (0.09)	
	Missing, n	108		81		9		11		209	

**Table 2:** Feedback from health professionals (HPs) and students on information consulted regarding situational relevance, cognitive impact, intention to use, and expected patient and health benefits (N=6447).

								HPs (n=3663)		Students (n=2784)
**Overall, did you search Stroke Engine for information on...**										
			General information on stroke rehabilitation, n (%)				894 (26.58)		693 (28.44)	
			Assessment approach, n (%)				1838 (54.64)		1228 (50.39)	
			Intervention approach, n (%)				309 (9.18)		143 (5.87)	
			e-Learning modules, n (%)				171 (5.08)		213 (8.74)	
			All of the above, n (%)				11 (0.33)		2 (0.08)	
			Other^a^, n (%)				141 (4.19)		158 (6.48)	
			Missing, n				299		347	
**Q1. Why did you do this search for information?^b^**										
			To address a clinical question, n (%)				990 (22.5)		300 (9.8)	
			To get new knowledge, n (%)				2063 (46.82)		2347 (76.67)	
			To share information with patient or family, n (%)				348 (7.9)		87 (2.84)	
			To share information with other HPs, n (%)				1005 (22.8)		327 (10.68)	
**Q2. Did you find relevant information that partially or completely met your objectives?**										
		Completely, n (%)						1741 (47.53)		1419 (50.97)
		Partially, n (%)						1762 (48.1)		1270 (45.62)
		No, n (%)						160 (4.37)		95 (3.41)
**Q3. What is the expected impact of this information on you or your practice?^b^**										
	**Practice changed or improved, n (%)**							1010 (27.57)		479 (17.2)
						Assessment approach	689 (68.22)		311 (64.9)	
						Treatment approach	198 (19.6)		115 (24)	
						Prognostic approach	52 (5.15)		21 (4.4)	
						Patient or family education	71 (7.03)		32 (6.7)	
	Learned something new, n (%)							1854 (50.61)		2080 (74.71)
	Information confirmed I was doing right, n (%)							1013 (27.65)		463 (16.63)
	I am reassured, n (%)							506 (13.81)		320 (11.49)
	Reminded of what I already knew, n (%)							750 (20.48)		374 (13.43)
	Problem with presentation of information, n (%)							27 (0.74)		15 (0.54)
	Disagree with information, n (%)							4 (0.11)		6 (0.22)
	Information potentially harmful, n (%)							2 (0.05)		4 (0.14)
**Q4. Did you (will you) use this information for a specific patient?**										
	Yes, n (%)							1503 (42.91)		496 (18.44)
	Possibly, n (%)							1237 (35.31)		1167 (43.4)
	No, n (%)							763 (21.78)		1026 (38.16)
	Missing, n							160		95
	**If yes, I will use the information to...^b^, n (%)**									
				Modify how I assess this patient			471 (31.3)		138 (27.8)	
				Modify how I treat this patient			288 (19.2)		95 (19.2)	
				Make a choice between options			295 (19.6)		108 (21.8)	
				Manage this patient			480 (31.9)		169 (34.1)	
				Be more certain about management			363 (24.2)		146 (29.4)	
				Better understand particular issue			398 (19.8)		151 (30.4)	
				Discuss with this patient			222 (14.8)		59 (11.9)	
				Discuss with other HPs			304 (20.2)		110 (22.2)	
				Influence this patient or HP regarding treatment			185 (12.3)		45 (9.1)	
**Q5. For this patient, did you observe (or do you expect) any health benefits as a result of applying this information?**										
	Yes, n (%)							623 (42.09)		203 (41.26)
	Possibly, n (%)							636 (42.97)		222 (45.12)
	No, n (%)							221 (14.93)		67 (13.62)
	Missing, n							2183		2292
	**If yes, I expect the benefits to..., n (%)**									
					Improve patient’s health status, functioning, or resilience		345 (55.4)		89 (43.8)	
					Prevent disease or worsening of disease		93 (14.9)		32 (15.8)	
					Avoid unnecessary or inappropriate treatment		183 (29.4)		46 (22.7)	
					Decrease patient’s worries		95 (15.2)		24 (11.8)	
					Increase patient’s or relatives’ knowledge		170 (27.3)		36 (17.7)	

^a^The *Other* response option was not specified.

^b^Multiple answers were allowed.

**Table 3:** Feedback from individuals with stroke and relatives on information consulted regarding situational relevance, cognitive impact, intention to use, and expected health benefits (N=187).

			Individuals with stroke (n=87)	Relatives (n=100)
**Overall, did you search Stroke Engine for information on...**				
	General information on stroke rehabilitation, n (%)		45 (59)	57 (62.6)
	Assessment approach, n (%)		11 (14)	19 (20.9)
	Intervention approach, n (%)		3 (4)	10 (11)
	e-Learning modules, n (%)		6 (8)	1 (1.1)
	All of the above, n (%)		1 (1)	0 (0)
	Other^a^, n (%)		10 (13)	4 (4.4)
	Missing, n		11	9
**Q1. Is this information relevant? n (%)**				
	Very relevant		34 (39)	50 (50)
	Relevant		37 (43)	39 (39)
	Somewhat relevant		13 (15)	8 (8)
	Not relevant (not the information I had hoped to find)		3 (3)	3 (3)
**Q2. Do you understand this information? n (%)**				
	Very well (I understood)		41 (47)	48 (48)
	Well		39 (45)	45 (45)
	Poorly		4 (5)	2 (2)
	Very poorly (I did not understand much)		3 (3)	5 (5)
**Q3. What do you think about this information?^b^, n (%)**				
	Teaches me something new		30 (34)	65 (65)
	Allows me to validate what I do or did		25 (29)	26 (26)
	Information reassures me		24 (28)	20 (20)
	Refreshes my memory		9 (10)	9 (9)
	Motivates me to learn		26 (30)	23 (23)
	I think there is a problem with the information		3 (3)	0 (0)
	I disagree with the information		0 (0)	1 (1)
	Information can have negative consequences		1 (1)	0 (0)
**Q4. Will you use this information?, n (%)**				
	Yes		77 (89)	92 (92)
	No		10 (11)	8 (8)
	**If yes, I will use the information to...^b^**			
		Help me better understand	42 (54.5)	57 (62)^c^
		Help me do something	10 (13)	22 (24)^c^
		Convinced me to do it	14 (18.2)	5 (5)^c^
		Do something in a different manner	15 (19.5)	9 (10)^c^
		Discuss with health professionals	15 (19.5)	25 (27)^c^
		Discuss with relatives and friends	14 (18.2)	28 (30)^c^
**Q5. Do you expect any benefit for you and your relative from using this information? n (%)**				
	Expect no benefits		12 (14)	3 (3)
	**This information will...**			
		Help improve health or well-being	35 (40)	54 (54)
		Help feel reassured	26 (30)	28 (28)
		Help prevent a problem or worsening of a problem	10 (11)	20 (20)
		Help handle a problem	16 (18)	20 (20)
		Prepare better for discussion with health professional	22 (25)	27 (27)
		Prepare better discussion with relatives	13 (15)	27 (27)
		More confident to make decision with health professional	17 (20)	13 (13)
		More confident to make decision with relatives	6 (7)	13 (13)

^a^The *Other* response option was not specified.

^b^Multiple answers were allowed.

^c^N=92.

### Situational Relevance

*Assessment approach* was the main topic searched by health professionals (1838/3364, 54.64%) and students (1228/2437, 50.39%), followed by general information on stroke rehabilitation (894/3364, 26.57% and 693/2437, 28.44%, respectively), which was the top-ranked topic for nearly two-thirds of the individuals with stroke (45/76, 59%) and their relatives (57/91, 63%), as reported in Tables 2 and 3.

Analysis of the free-text comments (n=950) indicated that although some respondents were looking at *how to obtain a test*—as illustrated by the following comments: “Interest in purchase of assessment” (ID3208, occupational therapist looking at the Activity Card Sort) and “Looking for a copy of the assessment” (ID3514, occupational therapist looking at the Wolf Motor Function Test)—others were searching for in-depth information that would allow for an accurate *interpretation of the test results*, as supported by comments such as “I am looking for the scores and the ranges of the scores and what the scores mean” (ID744, family member of a patient with a traumatic brain injury) and “I used the Trails A and B to test a patient and will use the information in this website to interpret the results” (ID1610, kinesiologist). Others were looking for information to assist them in their *clinical decision-making*; examples of comments include “Looking for more info in functional communication assessments in general” (ID1788, speech-language pathologist) and “I need the information found on your website to learn about available assessments as well as their psychometric properties to determine if the assessments are the best and most appropriate for the population I’m seeing” (ID2399, student).

Analysis of the comments from the individuals with stroke and their relatives showed that the information contributed to the *empowerment* of people regarding their own health and decisions and helped them to better *cope* with their situation as illustrated in the following comments: “I would like to prepare therapeutic materials to use while waiting for speech therapy to begin” (ID2701, relative); “I am a recent stroke victim, this information will very greatly help me in my recovery!” (ID3743, individual with stroke); “[Y]our article, which helped me to feel better about the future—especially since my stroke is cryptogenic” (ID4605, a health professional who had had a stroke); and “Better acceptance of a difficult diagnosis for the patient” (ID4820, relative).

Another case of situational relevance of information that emerged from the free-text comments related to searching information for *research purposes* as illustrated by these comments: “We are considering using the ARAT [Action Research Arm Test] as our primary outcome for a new data science research proposal” (ID245, physical therapist) and “I am using the CDT [Clock Drawing Test] in a research proposal” (ID452, student). In addition, a respondent commented as follows:

It’s really helpful, I am planning to use it for research.

ID1496, occupational therapist

Respondents also mentioned consulting the website for educational purposes because it is used as a *resource for teaching*: “I was looking for information to give my students” (ID396, occupational therapist), “I am an instructor and use the site frequently with OT [occupational therapy] students” (ID1238, occupational therapist), and “Using this information to teach students about aphasia assessments” (ID1978, speech-language pathologist). More specifically, other respondents mentioned using the website to *educate/inform clients*: “I use this resource to share with patients, students, and health professionals” (ID2125, librarian); and “[The Stroke Engine website] has become my first recommendation for patients and families who want to have access to reliable information regarding suggested or advertised stroke treatment modalities” (ID3974, rehabilitation medicine).

### Cognitive Impact

Of the health professionals and students who answered yes to the question about a change or an improvement in practice (Tables 2 and 3), approximately two-thirds related this change to the assessment approach (689/1010, 68.22% and 311/479, 64.9%, respectively). *Learning something new* was chosen by 74.71% (2080/2784) of the students, 65% (65/100) of the relatives, 50.61% (1854/3663) of the health professionals, and 34% (30/87) of the individuals with stroke, as exemplified by these free-text comments: “To enhance my knowledge” (ID1533, occupational therapist looking at the Motor-Free Visual Perception Test page), “Love to know more about this” (ID1574, student looking at the Motor-Free Visual Perception Test page), “I want to gain information regarding this topic” (ID1604, nurse looking at the General Health Questionnaire-28), “I will apply the knowledge I get from this questionnaire” (ID3857, physical therapist looking at the General Health Questionnaire-28), and “I don’t know this test yet and I’m looking into it to see with which patient I could use it” (ID5419, speech-language pathologist looking at the Boston Diagnostic Aphasia Examination).

### Intention to Use

The majority of the respondents reported an intention to use the information as reflected by the percentages of respondents who selected *no use*: 21.78% (763/3503) of the health professionals, 38.16% (1026/2689) of the students, 11% (10/87) of the individuals with stroke, and 8% (8/100) of their relatives (Tables 2 and 3). The free-text comments suggest that many of the respondents were searching for information to *refine a topic*: “Will help determine remediation strategies and possible problems at home upon discharge” (ID3986, occupational therapist looking at the Bells test), “I believe I will be able to target sedentary behaviour” (ID4078, physical therapist looking at a web-based aerobics course), and “Bookmarking this page for possible future reference once I graduate” (ID917, student looking at the home page). Respondents with *prior knowledge* who were already using an assessment were looking for information on administration procedures or interpretation of the scoring; for example, a respondent commented as follows:

[Information] confirmed improvement in language skills and appropriate home practice to continue on motor speech skills. It was helpful to have this on-line to allow me to analyze a report and score without having to return to the office to look at the manual.

ID3370, speech-language pathologist looking at the Western Aphasia Battery

Others already had the *intention to use a test* before accessing the website and were searching for information on how to obtain it (refer to the *how to obtain a test* subtheme in the *Situational Relevance* subsection).

Intention to use was high among all 4 subgroups, with the exception of the students: less than one-fifth (496/2689, 18.44%) answered yes to the question about their intention to use the information for a specific patient (Table 2). Indeed, many of the students were looking for information in the *context of a class assignment*: “Researching for assignment on right neglect” (ID289, student looking at the Bells test); “I am using this as a student to understand how depression can be assessed” (ID2002, student looking at the Beck Depression Inventory); “This information will help me on my board exam and when I get a job as an OTA [occupational therapy assistant]” (ID2654, student looking at the Executive Function Performance Test); “I am a PT [physical therapy] student, thank you for this clear explanation of the comb and razor test!” (ID3060, student looking at the Comb and Razor Test).

Although intention to use was high overall, the free-text comments allowed respondents to make suggestions regarding *how information provided could be improved* either in terms of *visual/format* or in *ease of finding***,** as illustrated by comments such as “Not helpful if I can’t download the PDF in Greek” (ID1294, pharmacist looking at the Mini Mental State Examination); “The Patient/family PDF link at the top of the page is linked to the wrong PDF, it is about electrical stimulation instead of positioning” (ID2543, physical therapist looking at Positioning); “I need a link to purchase” (ID2292, occupational therapist looking at the Motor-Free Visual Perception Test); “Make the presentation of information interesting, add pictures or other graphic that can catch people’s attention easier” (ID4523, student; the page visited was not recorded); and “Videos of the assessments are lacking” (ID4984, occupational therapist looking at the Berg Balance Scale). Others left comments asking for *more information* because the information provided was perceived as *incomplete*; for example, a respondent commented as follows:

Did not find any information. I have not found this website easy to use and prefer other websites.

ID2769, nurse; the page visited was not recorded

The other comments included “Need more information on population aim” (ID1201, student looking at the Chedoke Arm and Hand Inventory), “The info can be a bit more specific with more examples” (ID1732, student looking at the definition of intrarater reliability), “There was no information on the frequency of the test” (ID3566, nurse looking at the Clock Drawing Test), “The given information is helpful, but was expecting more detailed information as I am from medical field hence I was looking for depth information” (ID3746, physical therapist looking at the Glasgow Coma Scale), and “Scoring should be more elaborately explained” (ID4511, physical therapist; the page visited was not recorded).

Furthermore, a respondent provided the following comment regarding the information available on the website:

Incomplete information. Procedure required with more meaning.

ID4800, student; the page visited was not recorded

The respondents also used this opportunity to let us know which *topics they consider important enough to be added*; for example: “You make no mention of Personality changes nor Emotion Lability Episodes, both of which are very common consequences for Stroke survivors” (ID1266, individual with stroke on the *Contact us* page); and “I am trying to find more information on other perceptual difficulties such as construction or other spatial challenges” (ID3912, occupational therapist looking at Unilateral Spatial Neglect).

In addition, a respondent commented as follows:

It would be very useful to have a section for how to approach rehab for patients with Ataxia. Somewhere that summarises the basics of Ataxia management.

ID3188, occupational therapist looking at interventions by topic page

### Expected Patient and Health Benefits

The expectation that the use of the information would result in health benefits was relatively high (more than two-fifths of the respondents: 998/2159, 46.23%; refer to Tables 2 and 3) across all 4 subgroups. Improvement in health and well-being was the top-ranked category of expected benefits for all 4 subgroups, followed by the avoidance of unnecessary or inappropriate treatment for health professionals (183/623, 29.4%) and a feeling of being reassured for individuals with stroke (26/75, 35%) and their relatives (28/97, 29%). Examples of comments supporting expected benefits included “I want to know about benefits or uses of assistive devices for stroke patients” (ID1159, student looking at assistive devices). Other comments were related to the *benefits of using an assessment to provide feedback on improvements*, such as the following comment:

In selecting this assessment, which I’ve not previously used, I can complete information to provide a patient with post rehab scores to complement the pre rehab score on this test, completed at another facility. This will likely be beneficial to the client in knowing his achievements and also to the community team whom I am referring the patient to nearer his home.

ID2311, occupational therapist looking at the Occupational Therapy Adult Perceptual Screening Test

Other comments included “It was beneficial” (ID2916, student looking at the Boston Diagnostic Aphasia Examination) and “Using as an outcome measure after rehab to highlight improvement therefore may be of psychological benefit” (ID3016, occupational therapist looking at the Nine-Hole Peg Test).

### General Comments

Overall, the free-text comments were positive: “Thank you for raising health care standards!” (ID4180, occupational therapist looking at the National Institutes of Health Stroke Scale), “A useful summary of important information” (ID4563, clinical psychologist; the page visited was not recorded), “Thank you for this wealth of information that improves our practice!!” (ID5014, speech-language pathologist looking at the Bells test), and “Thank you for sharing your work you are always models for us!!!!” (ID5220, stroke pathway facilitator looking at the *Patients and Families* page). In addition, respondents provided the following comments:

Useful to have a variety of topics in one place. Information is brief but reasonably detailed so gives a good idea of what to do and not do.

ID4622, occupational therapist; the page visited was not recorded

Love Stroke Engine. Thank you for this resource!

ID4777, occupational therapist; the page visited was not recorded

Hello, I am a medical student and I was learning a course on how to measure motor impairments in people with disabilities, which led me to this site and I was able to find my happiness. Thank you.

ID5830, student looking at the Modified Ashworth Scale

## Discussion

### Principal Findings and Comparison With Prior Work

The main goal of this study was to document Stroke Engine users’ perceptions of situational relevance, cognitive impact, intention to use, and expected patient and health benefits regarding the information consulted. The main results relating to situational relevance showed that assessments (including selecting, obtaining, and interpreting results from a test) was the main topic searched by health professionals (1838/3364, 54.64%) and students (1228/2437, 50.39%), whereas general information on stroke rehabilitation was the top-ranked topic for nearly two-thirds of the individuals with stroke (45/76, 59%) and their relatives (57/91, 63%). Cognitive impact was characterized by *learning something new*. Intention to use was high (4590/6379, 71.95%) among respondents and varied in context (eg, refine a topic, research, class assignments, teaching, and education). Expected patient and health benefits such as improvement in health and well-being was the top-ranked category for all 4 subgroups, followed by an avoidance of unnecessary or inappropriate treatment for health professionals (183/623, 29.4%) and a feeling of being reassured for individuals with stroke (26/75, 35%) and their relatives (28/97, 29%). Overall, the results of this study highlighted the funnel pattern of the 4 levels of outcomes on information [24] where information can be relevant and have a cognitive impact but may not necessarily be used; conversely, information can be used but does not necessarily lead to health benefits. This illustrates information-related actions and subsequent outcomes regarding information users, including people with lived experience and their relatives. Indeed, although the information searched was deemed relevant and had a cognitive impact for 96.07% (6373/6634) of the respondents, intention to use dropped to 68.92% (4572/6634), and only 27.98% (1856/6634) of the respondents expected patient or health benefits. The drop relating to intention to use can be partially explained by a large representation in our sample of students (2784/6634, 41.97%), who typically use the information for class assignments. It may also be because practice change is a challenging process that requires more than access to knowledge [35,36]. In fact, a positive attitude toward scientific evidence was recently found to be the necessary and sufficient attribute to explain a high use of evidence-based practice among rehabilitation professionals [37]. We may hypothesize that the majority of our subgroup of health professionals (3663/6634, 55.22%) had a positive attitude toward scientific evidence because they initiated the search (they *pulled* the information), which is a different scenario than when the information is *pushed* to facilitate its implementation [38]. As shown by the free-text comments, many of the respondents were already users of the information and were searching for a link that would lead them to a specific assessment or searching for guidance on how to interpret a tool that they were already using in practice.

Interestingly, more than half of the health professionals and students searched for assessments rather than interventions (309/3364, 9.19% and 143/2437, 5.87%, respectively). The reasons for this are uncertain. They may already know how to intervene or be aware of the best treatment options. By contrast, it is possible that *they don’t know that they don’t know* (knowledge gap), and therefore they do not initiate a search for interventions, or perhaps they look into clinical practice guidelines for treatments. The generally agreed-upon time lag for scientific evidence to translate into practice is 17 years [39]. If we consider 2008 as the start of the rise in the number of publications of randomized controlled trials in stroke rehabilitation [14], this type of evidence-based knowledge can be arguably considered relatively recent. Looking at our results, we could interpret them as an incentive to further prioritize content on assessment on our website so that we may best meet users’ needs. However, this should not be at the expense of interventions because we anticipate that interventions will become an important topic as we strive to bridge the knowledge-to-practice gap [40].

The substantial underrepresentation of individuals with lived experience and their relatives in comparison with health professionals and students was striking, although the website is accessible to all. This may partly be due to the fact that, as scholarly practitioners [41], health professionals may facilitate translation and mobilization and therefore share health-related information with their clientele in practice. Health professionals have told us to provide printable PDF versions of relevant information from our website to their clientele. As such, nearly one-third (170/623, 27.3%) of the health professionals responded that they expected an increase in patients’ or relatives’ knowledge as a benefit, further supporting their role as a transmission belt of relevant information. It may also be that fewer individuals with stroke and their relatives filled in the IAM questionnaire because they may use our website as 1 resource among many others and also use it less formally compared with the other 2 subgroups.

This website was first created with the aim to narrow the knowledge-to-practice gap and support the evidence-based practice of health professionals. From its inception in 2008, the website has included lay summaries for people with lived experience and their relatives to help them to cope with the consequences of stroke. It was also designed to empower people with lived experience and their relatives to become a transmission belt and request specific interventions or, at the very least, open a dialogue with their therapists. Both quantitative results and free-text comments indicate that we are meeting this aim for individuals with stroke and their relatives who volunteered to complete the web-based survey. One challenge is to reach out to a greater audience of individuals with lived experience and their relatives. We might also question whether the current format and content are sufficient or how to improve both to meet the informational needs of a greater audience. In other words, what do nonrespondents think of the value of this content? What would be the best way or methods to give them a voice?

Finally, using crowdsourcing as a method for soliciting feedback enabled us to realize how important and relevant such a web resource can be not only for practice (its primary mandate) but also for education and research. Indeed, with students representing 41.97% (2784/6634) of the sample and as supported by the free-text comments, our website proved to be a premium resource to learn about stroke rehabilitation. One sector, however, that might be considered underrepresented would be the policy sector. Incidentally, we are aware that our website is used as a resource for national [42] and provincial [43] guidelines. Despite the website’s value for knowledge translation in practice, education, research, and policy, our biggest challenge is to secure recurrent funding to keep its content up to date and to further add innovations. Our hope is to incorporate artificial intelligence (AI; such as a chatbot) to facilitate an open evidence-based practice dialogue by allowing an easy exchange among scientific evidence (actual content of the website), tacit knowledge of health professionals, and experiential knowledge of people with lived experience and their relatives. However, to materialize our vision for incorporating AI, we would first need to secure funding to keep the actual website up to date. Indeed, research funding by national funding agencies proved to be of immense support when we first created this knowledge translation platform, but we do not have access to any funding programs to ensure its survival. Despite its relevance and usefulness for multiple stakeholders, most funding agencies view this resource as *infrastructure* and no longer as research. Given the perennial challenges of evidence-based practice and the intended purpose of knowledge translation, we have serious concerns about such a view. How can best practices be implemented in a sustainable manner without adequate funding? We invite discussion on how the absence of infrastructure funding for knowledge translation initiatives will affect patients and society at large.

### Strengths and Limitations

The use of crowdsourcing as a method of data collection can be seen as a strength because it allowed us to obtain valuable feedback from a large sample; however, it can also be seen as a limitation because we used convenience sampling [44]. As such, a first limitation concerns generalizability: the respondents may not be representative of all website users. A second limitation is the inclusion of a survey invitation pop-up window as suggested by users who could not easily find the link to the survey. Although its addition contributed to increasing our survey response rate, we wonder whether the pop-up window was appearing too early because some of the respondents commented about this. We know that too many pop-ups may irritate users by causing a distraction. Nevertheless, we do not know how this may have affected data collection, although visitors had the option to close the pop-up window and return later to complete the survey, which remained accessible at all times. The optional free-text comments to elicit concrete (practical) explanations or illustrations of survey responses is a strength of this study. Indeed, the IAM constitutes a reflexive learning method, thus justifying medical education credits in popular national programs, that stimulates thinking and constructive feedback. Although the option to provide free-text comments helped to collect feedback about areas for improvement, a third limitation lies in the fact that we were unable to analyze this feedback data according to the type of respondent.

### Future Directions

In sum, building on these results, first, we would recommend that knowledge translation resources that are comparable with Stroke Engine perform a similar evaluative process on a periodic basis, using a validated questionnaire such as the IAM. This tool enabled the retrieval of feedback not only on relevance of the information consulted but also on intention to use and expected health benefits, which is the essence of implementation sciences. Second, we would recommend including information in a *ready-to-use* format to minimize any potential barriers to implementation. Third, we would recommend exploring how AI can facilitate interactions among scientific evidence, tacit knowledge (through clinician users), and experiential knowledge (through people with lived experience). Fourth, we would recommend additional exploration as to how well AI can personalize the information searched, especially for people with lived experience and their relatives. Fifth and last—but probably the most important—we would recommend that research funding agencies reflect on current funding opportunities that by and large support new knowledge translation initiatives but do not account for a plan to ensure regular updates and sustained use.

### Conclusions

Valuable feedback on Stroke Engine was obtained in terms of its accessibility, relevance for informational needs and retrieval, accuracy, and applicability. The results of this study highlighted the funnel pattern of the 4 levels of outcomes regarding information where information can be relevant and have a cognitive impact but may not necessarily be used; conversely, information can be used but does not necessarily lead to health benefits. In this era of omnipresence of the internet for retrieving various types of information, including health-related information, it becomes of utmost importance to document how information posted on the web is perceived and received. The methods used in this study, including crowdsourcing through the IAM, allowed us to retrieve valuable feedback not only in terms of its accessibility, relevance for informational needs and retrieval, accuracy, and applicability but also, importantly, on the potential implementation of its evidence-based content in clinical practice and perceived expected impact for patients, their relatives, and health care professionals.

## Abbreviations

AI: artificial intelligence
IAM: information assessment method
KTA: knowledge-to-action

## Data Availability

As informed consent did not include a specific data-sharing agreement, the data sets analyzed during this study are available from the corresponding author on reasonable request.
